# Expert consensus on the development of a health-related questionnaire for the pediatric field of Korean medicine: a Delphi study

**DOI:** 10.1186/s12906-019-2796-x

**Published:** 2020-01-15

**Authors:** Jihong Lee, Sun Haeng Lee, Gyu Tae Chang

**Affiliations:** 1 Department of Pediatrics of Korean Medicine, Kyung Hee University Hospital at Gangdong, #892 Dongnam-ro, Gangdong-gu, Seoul, 05278 Republic of Korea; 2 Department of Clinical Korean Medicine, Graduate School, Kyung Hee University, Seoul, 02453 Republic of Korea; 3 Department of Pediatrics of Korean Medicine, Kyung Hee University Korean Medicine Hospital, Kyung Hee University Medical Center, Seoul, 02447 Republic of Korea

**Keywords:** Patient-reported outcome measure, Patient health questionnaire, Questionnaire development, Pediatrics, Child health, Children, Korean medicine, Delphi study, Consensus

## Abstract

**Background:**

Although a variety of patient-reported outcome measures (PROMs) for children have been developed, there is no pediatric PROM specific to Korean medicine (KM) that is validated by experts in the field. The aim of this study was to collate the opinions of specialists in KM pediatrics on the development of a generic PROM that can be used by Korean medical doctors to assess the health status of children.

**Methods:**

A three-round Delphi survey was conducted to determine the level of consensus on the development of a new PROM. Delphi questionnaires were sent by e-mail to 91 KM pediatricians on January 24, 2018. The Delphi questionnaire was composed of four sections: conceptualization, construction, items, and sources of content for a PROM. A nine-point Likert scale was used, and if more than two-thirds of the panels agreed or disagreed with a given sentence, they were considered to have reached a consensus. A draft of a PROM for the pediatric field of KM was developed in accordance with the preliminary conceptual framework.

**Results:**

Out of 91 experts, 18 finished three rounds of the Delphi survey. The experts reached a consensus on the necessity of a KM pediatric PROM for measuring various areas including child health, and using Likert scales with a recall period of 3 months. They also agreed on specific items and sources of content. A new draft of a health questionnaire for KM pediatrics was developed based on the Delphi consensus. It contains 44 items covering 7 domains: i) functions of the digestive system, ii) functions of the respiratory system, iii) mental functions, iv) skin functions, v) pain, vi) functions of the metabolic and endocrine systems, and vii) demographic details.

**Conclusions:**

This research represents the first step in developing a health questionnaire for the pediatric field of KM. The questionnaire can be used in clinical and research settings after verifying several types of validity and reliability.

## Background

Korean medicine (KM) has certain similarities with traditional Chinese medicine (TCM) and Kampo medicine in Japan; however, it was developed with considerable independent and distinctive features [1]. In the Republic of Korea, Korean medicine doctors (KMDs) and medical doctors obtain independent licenses and use different treatment methods. KMDs mainly use herbal medicine, acupuncture, moxibustion, and manual therapy for treating patients [2]. In 2016, 23,460 licensed KMDs were practicing at 14,142 KM clinics or KM hospitals in the Republic of Korea. Each year, approximately seven hundred and fifty KM students complete the six-year university course and take the required examinations for obtaining the national license [3]. The KM specialist training system was implemented in 1999 by the Ministry of Health and Welfare of Korea. After 4 years of training (one-year internship and a three-year residence course) in a KM hospital, KMDs are then qualified to take examinations for a specialist certification. The specialty subjects include pediatrics, gynecology, neuropsychiatry, ophthalmology-otorhinolaryngology-dermatology, acupuncture and moxibustion, internal medicine, rehabilitation medicine, and Sasang constitutional medicine [4]. Pediatrics is one of the specialties of KM, and it deals with the physiopathological characteristics and the treatment of ailments in children and adolescents from a KM perspective [5]. In 2016, there were 101 KM pediatricians in the Republic of Korea, and they accounted for 3.7% of all KMDs with specialist licenses [3].

One of the signature methods of approaching treatment in KM is to analyze, diagnose, and treat diseases from a holistic perspective. In KM, the human body is regarded as an organic whole, and when the internal organs are not functioning properly, it can manifest externally on the body surface. When Korean medicine doctors examine patients, they collect information about the whole body through four diagnostic methods including inspection, listening and smelling examinations, inquiry, and palpation; they then perform a comprehensive analysis of the signs and symptoms [6]. In addition to the four diagnostic methods that are based on the physician’s subjective judgment, KMDs use various questionnaires as reference materials for collecting clinical information; this method enables patients or caregivers to properly outline the details of their child’s health condition [7]. When a parent-reported questionnaire is used, herbal medicine treatment has been shown to be effective in improving stamina, appetite, digestion, and quality of sleep in children [8, 9]. In the case of TCM, clinical research evaluation guidelines recommend that researchers should evaluate aspects of a child’s overall health status such as digestion, appetite, urine, fatigue, sweat, or sleep, using TCM syndrome quantitative classification, in addition to the assessment of the main symptoms [10–12].

According to the US Food and Drug Administration (FDA), a patient-reported outcome measure (PROM) of treatment benefit is defined as data about a patient’s health condition that are reported directly by the patient without interpretation of the patient’s response by a clinician or anyone else [13]. When assessing children’s health status, it is frequently measured by informants, such as parents, teachers, or other caregivers. When the PROM of very young children cannot be reliably measured because of their developmental stages or language ability, perspective of the informants may be useful for determining the condition of the children [14–16].

Although a variety of PROMs for children have been developed [17–24], few studies have reported the development of a validated pediatric PROM that considers the overall health status of patients in a manner suitable for the unique methodologies of KM. When KMDs examine a pediatric patient, they identify the following items about systemic body functions, in addition to the main symptoms of the patient: sweat, heat and cold, headache, stomachache, eating, digestion, stool, urine, or sleep [5]. Although PROMs for young children tend to focus on physical functioning [5, 18, 19, 23], other existing measures are applied to measure a health related quality of life based on the World Health Organization’s definition of health, which is “a state of complete physical, mental, and social well-being and not merely the absence of disease” [18–24]. Therefore, it is necessary to apply a PROM that includes the items that KMDs need to know about a child’s physical health and the indicators that are important in KM treatment.

Out of all the KM pediatric literature in the Republic of Korea, “Five Viscera Weak Children Questionnaire (FVWCQ)” is one of the most frequently used questionnaires for measuring a child’s health status [25–30]. FVWCQ is a proxy-reported questionnaire containing from 5 [25–29] or 6 [30] domains, and from 30 [30] to 50 [25, 26, 28] or 55 [27, 29] items. It consists of items regarding circulatory, mental, neurological, digestive, respiratory, genitourinary, and metabolic problems. However, the FVWCQ has not been validated; items, domains, and response options are not equal to each other. There are certain challenges in answering questions that have several aspects in one item such as “sneezing, runny nose, and stuffy nose are frequent,” “the child has diarrhea or constipation,” or “the child has slow tooth development and frequently gets cavities.” Furthermore, there are questions that are confined to girls only, such as “In the case of a girl, is there vaginal discharge?” There are no indications for boys. In addition, there may be a methodological flaw in studies in which this questionnaire is used, since its reliability and validity have not been assessed [25–30]. Moreover, the FVWCQ has been used without users’ consensus. There is no reason why a questionnaire for KM pediatrics should be made in the framework of the five viscera among various theories of KM.

The aim of this study was to investigate the level of consensus of experts on a new pediatric PROM that is suited to the methodologies of KM, that can ease the monitoring and evaluation of the benefits of KM treatments in children. The Delphi questionnaire included the levels of consensus among experts with regard to concept, structure, items, and sources of content for a KM pediatric PROM. This article reports the findings from the Delphi survey and the preliminary framework and draft of a new pediatric PROM.

## Methods

### Design

#### The Delphi study

A three-round Delphi survey was conducted for this research. The Delphi method represents a relatively unbiased approach to decision-making method in health and social care and is used to determine the degree of consensus of experts on a topic [31]. The method consists of repeated surveys and feedback from experts without face-to-face meetings [32]. There is no restriction on the number of rounds that can be conducted [33], but two [34] or three rounds are most common [35]; the decision on the number of rounds required for any study is made at the discretion of the researcher [36]. Anonymity is guaranteed and this is what makes the Delphi method different from other consensus methods such as the nominal group technique [31]. Respondents can confirm their responses to the initial interrogation or reconsider their previous opinions. They may change their responses and can respond differently in light of replies from other respondents [32, 37]. Participants are provided with means, medians, standard deviation, and inter-quartile range, as well as collected opinions, as a reference for judgment [31].

#### Delphi questionnaire

The Delphi questionnaire was studied and examined by the research team. All of them are KMDs who completed six-year undergraduate courses and are specialists in KM pediatrics as well. One of them (CGT) is a professor of KM pediatrics at KM University with more than 20 years of clinical experience. The other two (LJ and LSH) are KMDs with more than 10 years of clinical experience.

The overall framework of the Delphi questionnaire was based on existing articles on PROM development and expert consensus [37, 38]. The Delphi questionnaire used in all rounds was composed of four sections that covered conceptualization, construction, items, and sources of content for the KM pediatrics questionnaire (Table 1). In the first section, panelists were asked whether the questionnaire should measure “body function” or “activities and participation” of children. The domains of “body functions” and “activities and participation” are based on the International classification of functioning, disability and health: children and youth version (ICF-CY). ICF-CY was developed by the World Health Organization and provides a common language for clinical health and research applications [39]. In section 2, issues covered the appropriateness of age range, response options, and recall period. In section 3, panelists were asked about the suitability of the items for the questionnaire. The proposed items were based on existing questionnaires [25] and KM pediatric textbooks [5]. In the fourth section, the panelists were asked about the sources of content for the questionnaire, such as interviews with children or parents, or existing PROMs. In addition, if the panelists had any other comments about the questionnaire in all rounds, they were able to write down their opinions in the blank spaces that were provided at the end of the Delphi questionnaire [see Additional file 1].

**Table 1:** Statements of the Delphi questionnaire

Section		Statements
Conceptualization		1. It is necessary to develop a standardized questionnaire that can be used by KMDs in KM treatment for children or in research.
		2. The <KM pediatric questionnaire> should measure various areas constituting pediatric health.
		3. The <KM pediatric questionnaire> should measure the body functions of children.
		4. The <KM pediatric questionnaire> should measure activities and participation of children.
		5. The <KM pediatric questionnaire> should be based on the <Five Viscera Weak Children Questionnaire>.
		6. The <KM pediatric questionnaire> should provide a total score.
		7. The <KM pediatric questionnaire> should provide a score for each area.
Construction	Age Range	1. A < KM pediatric questionnaire> should be developed for children aged 1–5.
		2. A < KM pediatric questionnaire> should be developed for children aged 6–9.
		3. A < KM pediatric questionnaire> should be developed for children aged 0–20.
	Response Options	4. It is appropriate to use Likert scales when responding to the <KM pediatric questionnaire>.
		5. It is appropriate to use visual analogue scales when responding to the <KM pediatric questionnaire>.
		6. It is appropriate to use dichotomic (yes/no) response options when responding to the <KM pediatric questionnaire>.
	Recall Period	7. It is appropriate to ask about the last 1 month.
		8. It is appropriate to ask about the last 3 months.
		9. It is appropriate to ask about the last 6 months.
		10. It is appropriate to ask about the last 1 year.
Items		How much do you agree that “the following items should be included in the questionnaire”? chills / cold hands and feet / hyperhidrosis / headache / dizziness and giddiness / arthralgia / chest discomfort / abdominal pain / vomiting / anorexia / thirst / diarrhea / constipation / frequent urination / sleeping disorder / fatigue / vitality / complexion / dry skin / frequent infections / rhinorrhea/nasal obstruction / epistaxis / being easily startled / anxiety / sensitivity
Sources of content	Sources of content	How important do you think the following materials are to the development of the questionnaire?
		1. Focus group interview (child)
		2. Focus group interview (parents)
		3. Focus group interview (experts)
		4. Existing pediatric PROM
	Literature references	5. KM pediatrics textbook
		6. TCM pediatrics textbook
		7. Conventional medicine pediatrics textbook
		8. Articles using <Five Viscera Weak Children Questionnaire>
		9. Articles on pediatric PROM

#### Participant selection

The research team targeted KM pediatricians and/or professors of KM pediatrics at KM universities in the Republic of Korea, because they mainly treat pediatric patients and will be the main users of the questionnaire. Participants were contacted via e-mail through the Society of Korean Medicine Pediatrics. Participants received full explanations of the study background, purpose, methods, and personal information protection in advance. Interested participants signed an informed consent form before joining the panel. The present study received ethical approval from the institutional review board of Kyung Hee University, Korean Medicine Hospital at Gangdong in the Republic of Korea (KHNMCOH 2017–12-008). The investigation adhered to the tenets of the Helsinki Declaration. Participants who did not submit the consent form were excluded from the study.

#### Sample size

The appropriate number of the participants to be included in a Delphi study is not specified [40]. The sample size was determined by taking into account the available expert resources and the scope of the issue [31, 41]. There was no consensus on the exact sample size; the range initially considered was from 15 to less than 50 [40]. If the sample size in the Delphi study is too large, a low response rate and difficulties in the summarizing process can be a potential problem [31, 40]. Considering that the number of KM pediatricians was 101 in 2016 [3], and due to time constraints, we considered that 15–20 participants would be suitable [42].

### Procedures

#### Data collection

In the first round, participants were given 2 weeks to complete the Delphi questionnaire. Participants were allowed to enter data into a Hangul program file (a word processor popular in the Republic of Korea for its specialized language writing abilities) so that they could be attached to an e-mail. If the subjects did not respond to or complete the questionnaire after more than a week, we considered that they were not willing to participate and made no attempt to contact them [33]. In the second and third rounds, we considered the participants familiar with the study method and the content of the Delphi questionnaire, and gave them 1 week to complete the questionnaires of each round.

#### Data management and analyses

The authors determined whether consensus had been reached based on the extent to which experts agreed on a given issue. Participants were asked to rank agreements or disagreements with statements on a scale of 1 to 9. One meant “totally disagree” and 9 “totally agree.” In the case of section 4, participants were asked about the importance of the sources of content, of which 1 meant “not important at all” and 9 “absolutely important.” In the first round, respondents were provided with reference materials to ensure that they could make informed decisions. The authors analyzed the responses for consensus and repeated the same questionnaire in the subsequent rounds. The second and third rounds provided the results (mean, median, standard deviation, and additional comments from other experts) of the previous round.

In the Delphi method, the definition of consensus among the participants is not established. The researchers determine how to measure the degree of agreement and the cut-off used to define the consensus [43]. Because the definition of the RAND group [44] is the most widely used, the authors adopted this definition. In a three-round survey, when more than two-thirds of the panels agreed or disagreed with a given sentence, they were considered to have reached a consensus. If they reached a consensus on a score of 7–9, it would be considered a “high agreement.” In case of an agreement score of 4–6, the author considered it a “middle agreement.” If they agreed on a score of 1–3, it was assumed a “low agreement.” If they failed to reach a consensus, it was considered “uncertain.” “Agreed” sentences were not presented in the next round [32, 37, 45].

#### Statistical analyses

Descriptive statistics were used and sociodemographic data were presented by frequencies (percentage) to describe the panelists. Microsoft Office Excel version 14.0 (Microsoft Corporation, Redmond, WA, USA) was used to analyze the results of the Delphi questionnaire.

### Preliminary conceptual framework and the questionnaire draft

A conceptual framework defines the concepts measured by a PROM in a diagram that describes the relationship between domains and items [13]. A preliminary conceptual framework based on the results of the Delphi consensus was developed. A questionnaire draft was designed based on this conceptual framework. In creating each question, the authors referred to articles on pediatric PROMs [15, 16, 18–24, 46]. A KM pediatrics textbook [5] and a conventional medicine pediatrics textbook [47] were also used as references, and were considered to be highly important by the respondents in the sources of content section in the Delphi survey questionnaire.

## Results

### Participants

**Fig. 1:**
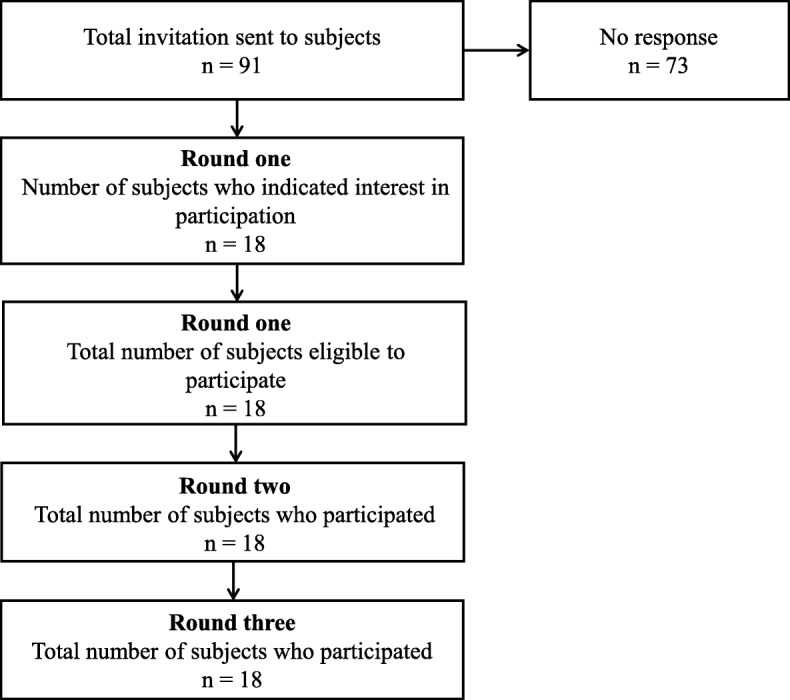
Flow of participation of the three-round Delphi procedure

Delphi questionnaires on PROM for KM pediatrics were sent by e-mail to 91 experts in KM pediatrics on January 24, 2018. The subjects were KM pediatric specialists that the research team could contact via e-mail. Eighteen out of the 91 subjects agreed to participate in the Delphi survey and replied to the first round Delphi questionnaire; the flow of the participation is shown in Fig. 1. All the KM pediatricians work in clinical care and 15 of them are professors of KM pediatrics at KM universities in the Republic of Korea. All 18 pediatricians completed the three rounds of the Delphi questionnaire. The sociodemographic characteristics of the panelists are shown in Table 2.

**Table 2:** Sociodemographic characteristics of the Delphi panelists (*n* = 18) involved in the development of the patient-reported outcome measures for the Korean medicine pediatric field

Factors	N (%)
Age (years)	
20–29	0 (0)
30–39	10 (55.6)
40–49	3 (16.7)
50–59	4 (22.2)
60–69	1 (5.5)
Sex	
Male	7 (38.9)
Female	11 (61.1)
Clinical experience (years)	
≤4	0 (0)
5–9	8 (44.4)
10–19	5 (27.8)
≥20	5 (27.8)
Level of healthcare facility of institution he/she is currently affiliated to	
Primary healthcare institution (Korean Medicine clinic)	3 (16.7)
Secondary healthcare institution (Korean Medicine hospital with 30 to 500 inpatient beds)	14 (77.8)
Other	1 (5.5)

### The Delphi result

#### Delphi – round one

The first round Delphi questionnaire contained 51 statements in 4 sections. In section 1, five of the seven items regarding conceptualization of the questionnaire were highly agreed upon (Table 3). Seventeen panelists (94.4%) highly agreed with the question of whether it is necessary to develop a standardized questionnaire for children’s KM treatment or research. All panelists (100%) agreed that “the questionnaire should measure various areas constituting pediatric health” and “the questionnaire should measure the body functions of children.” In addition, there was a strong consensus against the need to measure the “activities and participation” of children (14/18 or 77.8%) and to provide a score for each area (15/18 or 83.3%). Agreement was not reached on two statements (“the questionnaire should be based on the questionnaire of the ‘Five Viscera Weak Children Questionnaire’” and “the questionnaire should provide a total score”) and they were asked again in the next round.

**Table 3:**
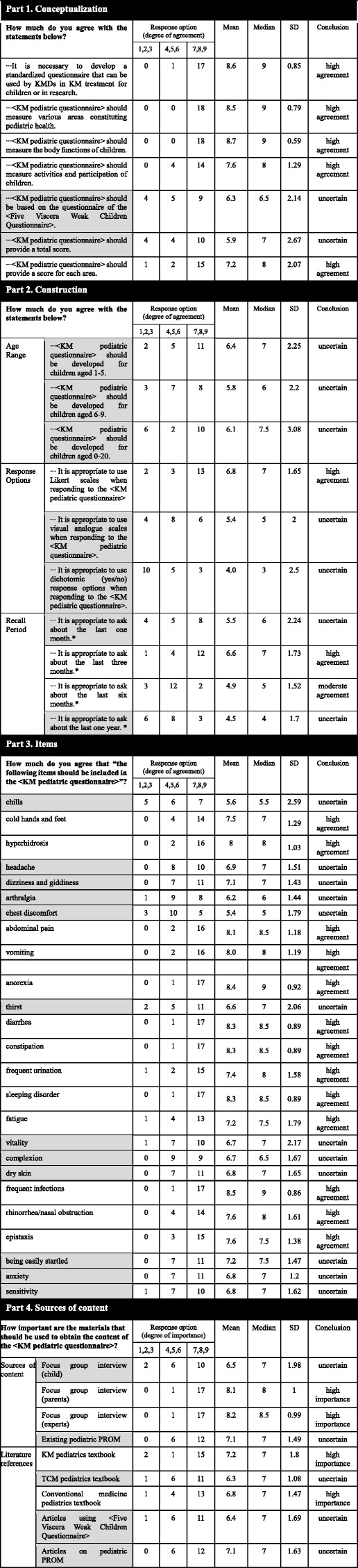
Participant responses from part 1 to part 4 (round one)

Greyed square means consensus was not reached on the statement. Non-greyed square means consensus was reached on the statement

Four statements marked with asterisk (*) represent statements in which one participant did not respond

*SD* Standard deviation, *KMD* Korean medicine doctor, *KM* Korean medicine; high agreement/importance: consensus on 7–9 points; middle agreement: consensus on 4–6 points; low agreement: consensus on 1–3 points; uncertain: failure to reach consensus, *PROM* Patient-reported outcome measure, *TCM* Traditional Chinese medicine

In section 2, the panel highly agreed on the use of Likert scales when responding to the questionnaire (13/18 or 72.2%). In section 2, one respondent did not respond to four statements asking for feedback on the recall period; this is noted in Table 3. He/she wrote in the blank spaces provided instead; he/she suggested that it would be better to divide the recall period according to stage of childhood, such as infancy and early childhood. In the same way, four statements with this missing response were considered to have been agreed on when more than two-thirds of the 17 participants agreed. There was a strong consensus to inquire about the last 3 months (12/17 or 70.6%) and moderate agreement to inquire about the last 6 months (12/17 or 70.6%). The panel disagreed on seven statements, including statements covering the age range; these statements were presented again in the second round.

In section 3, a high level of consensus was reached on 13 of 25 items in the first round such as cold hands and feet, hyperhidrosis, abdominal pain, vomiting, anorexia, diarrhea, constipation, frequent urination, sleeping disorder, fatigue, frequent infections, rhinorrhea/nasal obstruction, and epistaxis. Agreement was not reached on 12 items.

In section 4, the panelists agreed that interviews (parents, experts) are highly important. Regarding the literature references, the panel agreed that the KM pediatrics textbook and conventional medicine pediatrics textbook have a high degree of importance. There was no consensus on a focus group interview with children, on the existing pediatric PROM, and on several references as well (TCM pediatrics textbook, articles using the FVWCQ, and articles on pediatric PROM).

#### Delphi – round two

All 18 respondents in the first round responded in the second round. Twenty six statements were contained in the second round survey. In section 1, the panel did not agree on two statements in the second round (“the questionnaire should be based on the questionnaire of the FVWCQ”, “the questionnaire should provide a total score.”). For the age range in section 2, a moderate consensus was reached on the statement that a questionnaire should be developed for children aged 6–9 (13/18 or 72.2%). Agreement was not reached on the other six statements. In section 3, a high level of agreement was reached on two items: ‘vitality’ (13/18 or 72.2%) and ‘being easily startled’ (14/18 or 77.8%). A moderate agreement was reached on one item: ‘chest discomfort’ (14/18 or 77.8%), which means that out of 18 respondents, 14 indicated a degree of agreement from 4 to 6. Agreement was not reached on the other nine items. For the sources of content in the section 4, agreement was reached on the existing pediatric PROM being of high importance (16/18, 88.9%). For the literature references, agreement was reached on articles on pediatric PROM (14/18 or 77.8%, high importance) and TCM pediatrics textbook (13/18 or 72.2%, middle importance). There was still no agreement on statements on ‘focus group interview with children’ and ‘articles using FVWCQ’. Table 4 provides the responses from the participants in round two.

**Table 4:**
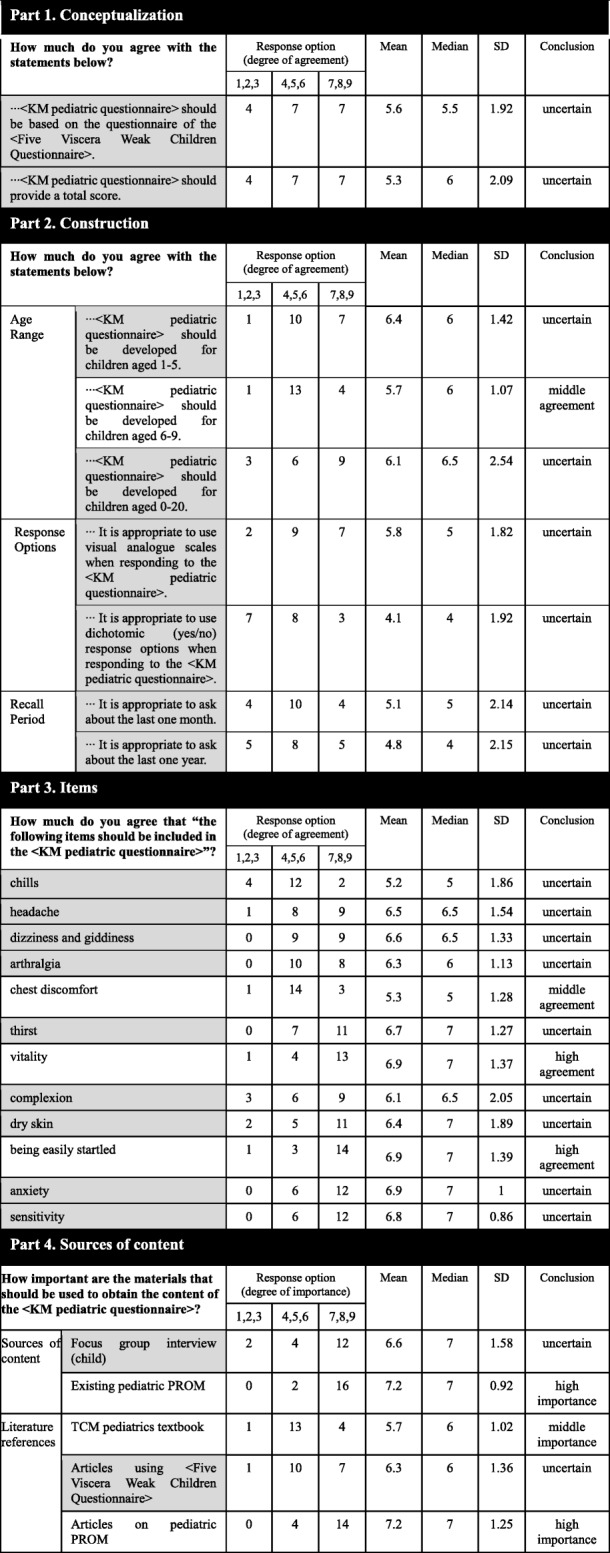
Participant responses from part 1 to part 4 (round two)

Greyed square means consensus was not reached on the statement. Non-greyed square means consensus was reached on the statement

*SD* Standard deviation, *KMD* Korean medicine doctor, *KM* Korean medicine; high agreement/ importance: consensus on 7–9 points; middle agreement/importance: consensus on 4–6 points; low agreement: consensus on 1–3 points; uncertain: failure to reach consensus; *PROM* Patient-reported outcome measure, *TCM* Traditional Chinese medicine

#### Delphi – round three

All 18 respondents in the second round responded in the third round. A total of 19 statements were contained in the third round survey. In section 1, agreement was still not reached on the same two statements. For the response options in section 2, agreement was reached on ‘use of dichotomic (yes/no) response options’ (15/18 or 83.3%, middle agreement). Furthermore, there was a moderate agreement on inquiring about the last month (14/18 or 77.8%). In section 3, consensus was still not reached on the nine items that the panel did not agree on in the second round. In section 4, agreement was not reached on statements on the importance of ‘focus group interview with children’ and ‘articles using FVWCQ’. The response from the participants in round three are presented in Table 5.

**Table 5:**
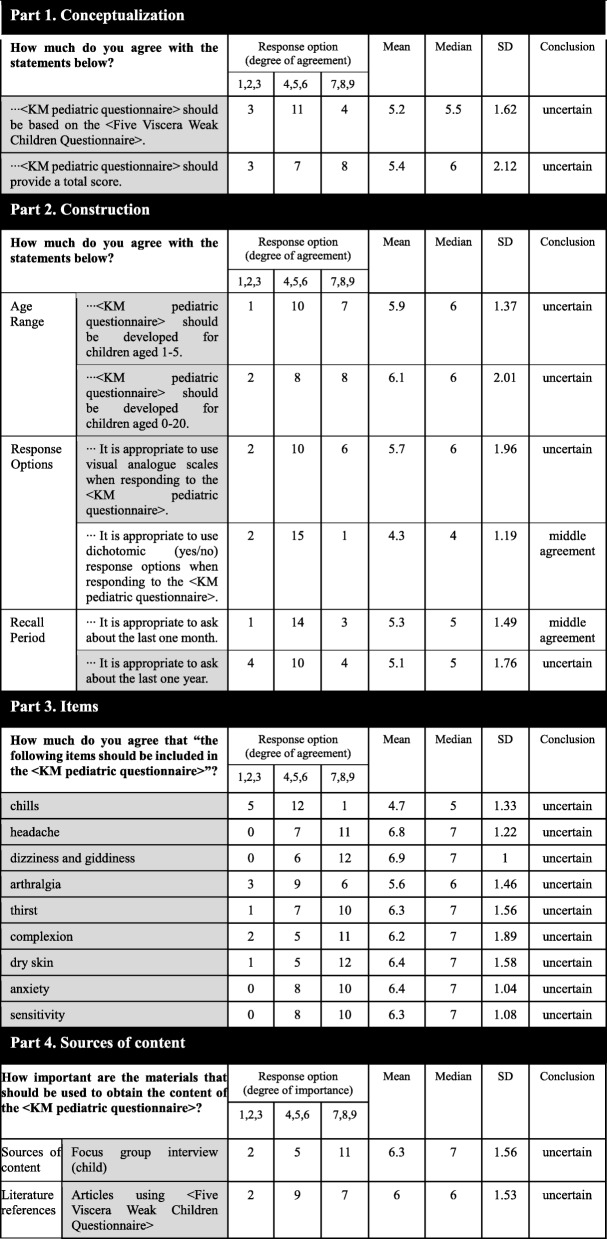
Participant responses from part 1 to part 4 (round three)

Greyed square means consensus was not reached on the statement. Non-greyed square means consensus was reached on the statement

*SD* Standard deviation, *KMD* Korean medicine doctor, *KM* Korean medicine; high agreement: consensus on 7–9 points; middle agreement: consensus on 4–6 points; low agreement: consensus on 1–3 points; uncertain: failure to reach consensus, *PROM* Patient-reported outcome measure, *TCM* Traditional Chinese medicine

### Preliminary conceptual framework and draft of questionnaire

**Fig. 2:**
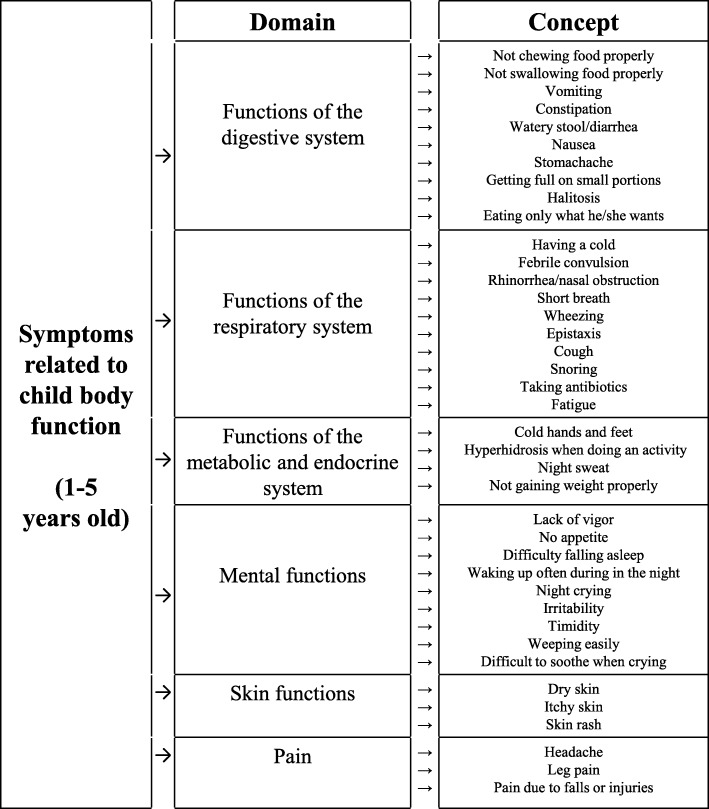
Preliminary conceptual framework for the KM pediatric questionnaire

The authors developed a preliminary conceptual framework based on the Delphi survey results (Fig. 2). As suggested by the Delphi panel’s additional comments, questionnaires needed to be developed individually for each age group, so the authors decided to develop a proxy-reported outcome measure for children aged 1–5 years for starters. This age group was found to be most frequent of all pediatric ages in the KM pediatric outpatient clinic [48–50]. The draft of the new questionnaire, based on the conceptual framework, consists of 7 domains and 44 items. In addition, the questionnaire includes five sociodemographic questions for the respondents and is to use a 5-point Likert scale [see Additional file 2].

## Discussion

This study presents the results of the development of a PROM for KM pediatrics, which assesses a child’s health status in a manner specific to KM, using the Delphi method. The Delphi survey was conducted to investigate the consensus of experts on the new PROM. Results from the Delphi survey revealed that 17 experts (94.4%) thought that it was necessary to develop a standardized PROM for KM pediatrics. However, the experts could not reach an agreement as to whether a new PROM should be based on the FVWCQ, which was frequently used in the KM pediatrics field [25–30]. When the authors asked the experts to give their opinions on the new PROM, four experts replied that the new questionnaire did not necessarily have to be in the same format as the FVWCQ, and after reviewing it, certain items may be used as needed. One expert replied that because the items of the FVWCQ were artificially developed to fit the five viscera, it was not necessary to refer to a new PROM. Because the FVWCQ has been used without expert consensus or validation [51, 52], if there are any useful items, they can be used restrictively only after critical review from experts.

Concerning the age range, the experts did not highly agree on any sentence in that regard. The experts suggested that separate questionnaires for all childhood age groups and adolescence are needed and should be developed to cover items according to the growth and development of each childhood and adolescence period. After a discussion session, the authors’ decision was to develop a questionnaire for children aged 1–5 years first, which according to a survey is the most common age group of all patients in the outpatient clinics of KM pediatrics [48–50]. It is known that there is no clear evidence of the validity or reliability of self-reporting measures in children younger than 5 years; it is necessary to use the observational reports of parents or other caregivers and special clinical measures when assessing the health status of children in this age range [14]. PROMs covering children 5 years old or younger commonly provide proxy-reported versions [14, 17, 18, 21, 24]. Therefore, the authors developed a PROM that utilizes parent-proxy reported outcome measures. Furthermore, in order to fit the characteristics of a PROM for younger children (younger than 5 years), the questionnaire consisted of items regarding “body function” rather than “activities and participation” [18, 19, 22, 46].

Regarding response options, 77.2% of the experts agreed that the Likert scales are appropriate for a PROM for KM pediatrics. Likert scales are considered appropriate for a pediatric PROM and a considerable number of PROMs have applied this scale [14]. In addition, a cognitive interview study established that a 5-point Likert scale is not difficult to understand even in the case of young children as young as 8 years old [53]. In accordance with the recommendations of the International Society for Pharmacoeconomics and Outcomes Research guideline [14], a follow-up cognitive interview was conducted to determine whether a 5-point Likert scale is appropriate for the questionnaire.

The recall period was determined to be 3 months, according to the experts’ consensus (70.6%, high agreement). In the open-ended questionnaire, two experts suggested applying different recall periods for each item. The recall period is controversial because the longer the recall period, the more likely it is to recall errors. Therefore, possible errors should be investigated through future interviews with parents.

The questionnaire draft consisted of items the experts agreed on (i.e., cold hands and feet, hyperhidrosis, abdominal pain, vomiting, diarrhea) and items extracted from textbooks (halitosis, cough, snore, wheezing, short breath, night crying) based on the results of the Delphi study. For clear communication, the item terms and domain classifications followed the ICF-CY. The ICF-CY was developed to be used by clinicians, family members, and researchers to document characteristics of children’s health [39] and has been used to identify the properties of PROMs [17]. For example, an item such as sleeping disorder that the experts agreed on in the Delphi study was divided into “difficulty falling asleep” and “waking up often during the night,” with reference to the ICF-CY.

Experts recommended a diversity of symptoms to be included. However, the purpose of this questionnaire was not to identify all the symptoms that may appear in children aged 1–5. The items of the questionnaire were intended to be the symptoms common to the physical functions of children and sensitive enough to detect change [13] according to KM treatment modalities. Items that a clinician should directly evaluate were excluded whereas items to be assessed by inquiry were included. Items that parents could directly observe and describe were also included. In addition, the authors focused on making it easy for symptoms to be checked at the clinics or in clinical researches. Items such as cognition, language, motor, or psychosocial development were not included in the questionnaire because these items were judged particularly important by the use of a disease-specific assessment tool and examination by a specialist.

The draft of the questionnaire will be modified through an iterative process into a more appropriate questionnaire, and then the next step is the content validation of the questionnaire. Content validity should first be established among several types of validities in the development of a questionnaire. Such validity is evidence that a PROM evaluates the concept of interest data from qualitative research, and that the domains and items are appropriate. Focus group interviews, individual interviews, or cognitive interviews are recommended for content validity [13].

The draft of the KM pediatric questionnaire is a generic measure of physical health of preschool children, which was developed as a monitoring and evaluation scale. It can be administered by specialists in the different fields of health, including KMDs. It can be used for healthy and ill children aged 1 to 5 years, and can be filled in by the parents. This questionnaire consists of 44 items covering 7 domains: i) functions of the digestive system (10 items), ii) functions of the respiratory system (10 items), iii) mental functions (9 items), iv) skin functions (3 items), v) pain (3 items), vi) functions of the metabolic and endocrine systems (4 items), and vii) demographic details (5 items). This questionnaire assesses the frequency of signs and symptoms. A 5-point Likert scale was applied on the formal items (0 means “never a problem”; 1 means “almost never a problem”; 2 means “sometimes a problem”; 3 means “often a problem”; 4 means “almost always a problem”). The recall period was 3 months. A higher score indicated that the child had many symptoms associated with physical functioning.

This questionnaire was drafted in accordance with FDA guidelines, based on expert agreement for the first time in the field of KM pediatrics [13]. The questionnaire contains items considered important in KM that are not included in other PROMs, for example, items such as cold hands and feet, halitosis, hyperhidrosis when doing activity, and night sweat.

A potential limitation of this study was that only 18 experts were included in the Delphi survey. Because the number of experts was small, there may be concepts and items that were not sufficiently included. This is due to the nature of Delphi research, which requires anonymity and voluntary participation in the recruitment process [33]. In addition, the nature of the research method did not allow free face-to-face discussions. However, the Delphi method is known to be a quick and reasonably easy way to carry out a survey, particularly via e-mail [37]. Instead, there was a blank space for an open-ended answer in the Delphi questionnaire, and the participants were able to express their opinions freely. The authors collected it as a reference for the next round. However, there can be fewer exchanges of opinions in this method than with a direct meeting. In round 3, there was not much change in the opinions of the panels, and the experts agreed on 2 among the 19 statements. Another limitation is the narrow diversity of respondents. Of 18 panelists, 15 were professors of KM pediatrics at university, and they had expertise in the pediatrics of KM. However, they might view the questionnaire differently from clinicians who are not academics. There is a limitation that the opinions of KMDs working at the primary healthcare institutions have not been fully collected. Further limitation is that there is an unavoidable uncertainty that this PROM may have because it is a proxy-reported outcome measure. When a parent reports on a measure, the items are interpreted, and the informant intervenes in the subjective health status of a child [14].

In this study, the Delphi survey was used to construct the framework of a questionnaire for KM pediatrics. Consensus was reached by the Delphi panel on the concept and construction of the questionnaire. This is an important step in the development of a pediatric PROM for KM. The next step will be individual interviews and cognitive interviews with parents for evidence of content validity. Further support of validity and reliability by psychometric evaluation such as construct validity, internal consistency, and test-retest reliability is required. This questionnaire may be used as objective data in the medical examination of children in KM clinics, and the quality and reliability of the KM treatment may be improved through a validated assessment scale. In addition, once the validity and reliability of the questionnaire are established in the future, it may improve the quality of research in KM pediatrics, and enable better communication with healthcare professionals.

## Conclusions

This research represents the first step in developing a PROM for KM pediatrics. The draft of the new questionnaire is a proxy-reported outcome measure and consists of 44 items and 7 domains, and a 3-month recall period. The content validity of this PROM will be confirmed later through interviews with parents; future psychometric evaluations of this PROM will make it a more reliable and valid questionnaire. If the PROM goes through a rigorous validation process, it will become a dependable method of accurately catching important and significant symptoms indicators in KM treatment. Moreover, it will be possible to get a better understanding of the health status of children.

## Supplementary information

**Additional file 1.** Delphi survey questionnaire.

**Additional file 2.** The draft of the Korean Medicine pediatric questionnaire.

## Data Availability

Not applicable.

## Abbreviations

FDA: Food and Drug Administration
FVWCQ: Five Viscera Weak Children Questionnaire
ICF-CY: International classification of functioning, disability and health: children and youth version
KM: Korean medicine
KMD: Korean medicine doctors
PROM: patient-reported outcome measure
TCM: traditional Chinese medicine
