# Supplementary material for: Expert consensus on the development of a health-related questionnaire for the pediatric field of Korean medicine: a Delphi study
Source: BMC Complement Med Ther. 2020 Jan 15;20:10. doi: 10.1186/s12906-019-2796-x (PMC7076900; doi:10.1186/s12906-019-2796-x)
Supplement: Supplementary file 1 — Additional file 1. Delphi survey questionnaire. [file 12906_2019_2796_MOESM1_ESM.docx]

**Additional file 1.**

**A Delphi survey for the development of the**

**<Korean Medicine pediatric questionnaire>**

| We would appreciate it if you could share your opinions about a questionnaire for Korean Medicine (KM) pediatrics with us. Please read the attached reference materials for the Delphi questionnaire and indicate your replies to the statements in the questionnaire provided. The Delphi questionnaire used in all rounds is composed of four sections that cover conceptualization, construction, items, and sources of content for the KM pediatric questionnaire. Please rank agreements or disagreements with statements on a scale of 1 to 9. 1 means “totally disagree” and 9 means “totally agree.” In the case of section 4, we are inquiring about your opinion on the importance of the sources of content, of which 1 means “not important at all” and 9 “absolutely important.” If you have any other comments, please write them in the Remarks column. The last chapter contains four demographic questions. Thank you for your participation. |
| --- |

**Part 1. Conceptualization of the KM pediatric questionnaire**

| Please indicate your opinion on the KM pediatric questionnaire by ticking one box only for each statement. | **Disagree ------------ Degree of agreement ---------- Agree** | | | | | | | | |
| --- | --- | --- | --- | --- | --- | --- | --- | --- | --- |
|  | **1**  Totally disagree | **2** | **3** | **4** | **5** | **6** | **7** | **8** | **9**  Totally agree |
| **Q1.** It is necessary to develop a standardized questionnaire that can be used by KMDs in KM treatment for children or in research. |  |  |  |  |  |  |  |  |  |
| **Q2.** <KM pediatric questionnaire> should measure various areas constituting pediatric health. |  |  |  |  |  |  |  |  |  |
| **Q3.** <KM pediatric questionnaire> should measure the body functions of children. |  |  |  |  |  |  |  |  |  |
| **Q4.** <KM pediatric questionnaire> should measure the activities and participation of children. |  |  |  |  |  |  |  |  |  |
| **Q5.** <KM pediatric questionnaire> should be based on the <Five Viscera Weak Children Questionnaire>. |  |  |  |  |  |  |  |  |  |
| **Q6.** <KM pediatric questionnaire> should provide a total score. |  |  |  |  |  |  |  |  |  |
| **Q7.** <KM pediatric questionnaire> should provide a score for each area. |  |  |  |  |  |  |  |  |  |
| **<Is there any other additional comment regarding the conceptualization of the KM pediatric questionnaire?>** | | | | | | | | | |

**Part 2. Construction of the KM pediatric questionnaire**

| Construction | Please indicate your opinion on the questionnaire for Korean Medicine pediatrics by ticking one box only for each statement. | **Disagree ---------- Degree of agreement --------- Agree** | | | | | | | | |
| --- | --- | --- | --- | --- | --- | --- | --- | --- | --- | --- |
|  |  | **1**  Totally disagree | **2** | **3** | **4** | **5** | **6** | **7** | **8** | **9**  Totally agree |
| Age Range | **Q1.** <KM pediatric questionnaire> should be developed for children aged 1-5. |  |  |  |  |  |  |  |  |  |
|  | **Q2.** <KM pediatric questionnaire> should be developed for children aged 6-9. |  |  |  |  |  |  |  |  |  |
|  | **Q3.** <KM pediatric questionnaire> should be developed for children aged 0-20. |  |  |  |  |  |  |  |  |  |
| Response Options | **Q4.** It is appropriate to use Likert scales when responding to the <KM pediatric questionnaire> |  |  |  |  |  |  |  |  |  |
|  | **Q5.** It is appropriate to use visual analogue scales when responding to the <KM pediatric questionnaire>. |  |  |  |  |  |  |  |  |  |
|  | **Q6.** It is appropriate to use dichotomic (yes/no) response options when responding to the <KM pediatric questionnaire>. |  |  |  |  |  |  |  |  |  |
| Recall Period | **Q7.** It is appropriate to ask about the last one month. |  |  |  |  |  |  |  |  |  |
|  | **Q8.** It is appropriate to ask about the last three months. |  |  |  |  |  |  |  |  |  |
|  | **Q9.** It is appropriate to ask about the last six months. |  |  |  |  |  |  |  |  |  |
|  | **Q10.** It is appropriate to ask about the last one year. |  |  |  |  |  |  |  |  |  |
| **<Is there any other additional comment regarding the construction of the Korean Medicine pediatric** **questionnaire?>** | | | | | | | | | | |

**Part 3. Items for the Korean Medicine pediatric** **questionnaire**

| Please indicate your opinion on the Korean Medicine pediatric questionnaire by ticking one box only for each statement. | | **Disagree ------------- Degree of agreement -------------- Agree** | | | | | | | | |
| --- | --- | --- | --- | --- | --- | --- | --- | --- | --- | --- |
|  |  | **1**  Totally disagree | **2** | **3** | **4** | **5** | **6** | **7** | **8** | **9**  Totally agree |
| How much do you agree that “the following items should be included in the questionnaire”? | **1.** chills |  |  |  |  |  |  |  |  |  |
|  | **2.** cold hands and feet |  |  |  |  |  |  |  |  |  |
|  | **3.** hyperhidrosis |  |  |  |  |  |  |  |  |  |
|  | **4.** headache |  |  |  |  |  |  |  |  |  |
|  | **5.** dizziness and giddiness |  |  |  |  |  |  |  |  |  |
|  | **6.** arthralgia |  |  |  |  |  |  |  |  |  |
|  | **7.** chest discomfort |  |  |  |  |  |  |  |  |  |
|  | **8.** abdominal pain |  |  |  |  |  |  |  |  |  |
|  | **9.** vomiting |  |  |  |  |  |  |  |  |  |
|  | **10.** anorexia |  |  |  |  |  |  |  |  |  |
|  | **11.** thirst |  |  |  |  |  |  |  |  |  |
|  | **12.** diarrhea |  |  |  |  |  |  |  |  |  |
|  | **13.** constipation |  |  |  |  |  |  |  |  |  |
|  | **14.** frequent urination |  |  |  |  |  |  |  |  |  |
|  | **15.** sleeping disorder |  |  |  |  |  |  |  |  |  |
|  | **16.** fatigue |  |  |  |  |  |  |  |  |  |
|  | **17.** vitality |  |  |  |  |  |  |  |  |  |
|  | **18.** complexion |  |  |  |  |  |  |  |  |  |
|  | **19.** dry skin |  |  |  |  |  |  |  |  |  |
|  | **20.** frequent infections |  |  |  |  |  |  |  |  |  |
|  | **21.** rhinorrhea/nasal obstruction |  |  |  |  |  |  |  |  |  |
|  | **22.** epistaxis |  |  |  |  |  |  |  |  |  |
|  | **23.** being easily startled |  |  |  |  |  |  |  |  |  |
|  | **24.** anxiety |  |  |  |  |  |  |  |  |  |
|  | **25.** sensitivity |  |  |  |  |  |  |  |  |  |
| **<Is there any other additional comment regarding the items of the Korean Medicine pediatric** **questionnaire?>** | | | | | | | | | | |

**Part 4. Sources of content for the Korean Medicine pediatric** **questionnaire**

| Please indicate your opinion on the Korean Medicine pediatric questionnaire by ticking one box only for each statement.  How important do you think the following materials are to the development of the questionnaire? | | **Not important----Degree of importance----Important** | | | | | | | | |
| --- | --- | --- | --- | --- | --- | --- | --- | --- | --- | --- |
|  |  | 1  not important at all | 2 | 3 | 4 | 5 | 6 | 7 | 8 | 9  absolutely important |
| Sources of content | **1-1.** Focus group interview (child) |  |  |  |  |  |  |  |  |  |
|  | **1-2.** Focus group interview (parents) |  |  |  |  |  |  |  |  |  |
|  | **1-3.** Focus group interview (experts) |  |  |  |  |  |  |  |  |  |
|  | **1-4.** Existing pediatric PROM |  |  |  |  |  |  |  |  |  |
| Literature references | **2-1.** KM pediatrics textbook |  |  |  |  |  |  |  |  |  |
|  | **2-2.** TCM pediatrics textbook |  |  |  |  |  |  |  |  |  |
|  | **2-3.** Conventional medicine pediatrics textbook |  |  |  |  |  |  |  |  |  |
|  | **2-4.** Articles using <Five Viscera Weak Children Questionnaire> |  |  |  |  |  |  |  |  |  |
|  | **2-5.** Articles on pediatric PROM |  |  |  |  |  |  |  |  |  |
| **<Is there any other additional comment regarding the sources of contents for the Korean Medicine pediatric** **questionnaire?>** | | | | | | | | | | |

| **DEMOGRAPHICS** Please provide the appropriate number. | | **Answer** |
| --- | --- | --- |
| **Q1.** What is your age group? | ① 20-29 years ② 30-39 years  ③ 40-49 years ④ 50-59 years  ⑤ 60 years or over |  |
| **Q2.** What is your gender? | ① Male ② Female |  |
| **Q3.** What is your affiliated institution? | ① Korean Medicine clinic (primary healthcare institution)  ② Korean Medicine hospital with 30 to 500 inpatient beds (secondary healthcare)  ③ Other |  |
| **Q4.** What are the total numbers of years you have worked as a Korean medicine doctor?  (year of clinical experience) | ① ≤4  ② 5-9  ③ 10-19  ④ ≥20 |  |
| **Thank you for your valuable time and input in this survey** | | |
