# Supplementary material for: Expert consensus on the development of a health-related questionnaire for the pediatric field of Korean medicine: a Delphi study
Source: BMC Complement Med Ther. 2020 Jan 15;20:10. doi: 10.1186/s12906-019-2796-x (PMC7076900; doi:10.1186/s12906-019-2796-x)
Supplement: Supplementary file 2 — Additional file 2. The draft of the Korean Medicine pediatric questionnaire. [file 12906_2019_2796_MOESM2_ESM.docx]

| **[Draft]** Korean Medicine Pediatric Questionnaire for Preschool Children | | | | |
| --- | --- | --- | --- | --- |
| Questionnaire for caregivers on the physical health of children aged 1-5 | | | | |
| ▶ please remark ○ on the state of your child for the last 3 months including the present. | | | | |
| **Please indicate your answer for each question by choosing one of the following five options.**  **How often does your child experience the following symptoms?** | | | | |
| **Never**  ⓪ | **Seldom**  **①** | **Quite often**  **②** | **Very often**  **③** | **Always**  **④** |

| **Functions of the digestive system** | | | | | | |
| --- | --- | --- | --- | --- | --- | --- |
| **1** | Not chewing food properly | ⓪ | **①** | **②** | **③** | **④** |
| **2** | Not swallowing food properly | ⓪ | **①** | **②** | **③** | **④** |
| **3** | Vomiting | ⓪ | **①** | **②** | **③** | **④** |
| **4** | Constipation | ⓪ | **①** | **②** | **③** | **④** |
| **5** | Watery stool/diarrhea | ⓪ | **①** | **②** | **③** | **④** |
| **6** | Nausea | ⓪ | **①** | **②** | **③** | **④** |
| **7** | Stomachache | ⓪ | **①** | **②** | **③** | **④** |
| **8** | Getting full on small portions | ⓪ | **①** | **②** | **③** | **④** |
| **9** | Halitosis | ⓪ | **①** | **②** | **③** | **④** |
| **10** | Eating only what he/she wants | ⓪ | **①** | **②** | **③** | **④** |

| **Functions of the respiratory system** | | | | | | |
| --- | --- | --- | --- | --- | --- | --- |
| **1** | Having a cold | ⓪ | **①** | **②** | **③** | **④** |
| **2** | Febrile convulsion | ⓪ | **①** | **②** | **③** | **④** |
| **3** | Rhinorrhea/nasal obstruction | ⓪ | **①** | **②** | **③** | **④** |
| **4** | Short breath | ⓪ | **①** | **②** | **③** | **④** |
| **5** | Wheezing | ⓪ | **①** | **②** | **③** | **④** |
| **6** | Epistaxis | ⓪ | **①** | **②** | **③** | **④** |
| **7** | Cough | ⓪ | **①** | **②** | **③** | **④** |
| **8** | Snoring | ⓪ | **①** | **②** | **③** | **④** |
| **9** | Taking antibiotics | ⓪ | **①** | **②** | **③** | **④** |
| **10** | Fatigue | ⓪ | **①** | **②** | **③** | **④** |

| **Functions of the metabolic and endocrine system** | | | | | | |
| --- | --- | --- | --- | --- | --- | --- |
| **1** | Cold hands and feet | ⓪ | **①** | **②** | **③** | **④** |
| **2** | Hyperhidrosis when doing an activity | ⓪ | **①** | **②** | **③** | **④** |
| **3** | Night sweat | ⓪ | **①** | **②** | **③** | **④** |
| **4** | Not gaining weight properly | ⓪ | **①** | **②** | **③** | **④** |

| **Mental functions** | | | | | | |
| --- | --- | --- | --- | --- | --- | --- |
| **1** | Lack of vigor | ⓪ | **①** | **②** | **③** | **④** |
| **2** | No appetite | ⓪ | **①** | **②** | **③** | **④** |
| **3** | Difficulty falling asleep | ⓪ | **①** | **②** | **③** | **④** |
| **4** | Waking up often in the night | ⓪ | **①** | **②** | **③** | **④** |
| **5** | Night crying | ⓪ | **①** | **②** | **③** | **④** |
| **6** | Irritability | ⓪ | **①** | **②** | **③** | **④** |
| **7** | Timidity | ⓪ | **①** | **②** | **③** | **④** |
| **8** | Weeping easily | ⓪ | **①** | **②** | **③** | **④** |
| **9** | Difficult to soothe when crying | ⓪ | **①** | **②** | **③** | **④** |

| **Skin functions** | | | | | | |
| --- | --- | --- | --- | --- | --- | --- |
| **1** | Dry skin | ⓪ | **①** | **②** | **③** | **④** |
| **2** | Itchy skin | ⓪ | **①** | **②** | **③** | **④** |
| **3** | Skin rash | ⓪ | **①** | **②** | **③** | **④** |

| **Pain** | | | | | | |
| --- | --- | --- | --- | --- | --- | --- |
| **1** | Headache | ⓪ | **①** | **②** | **③** | **④** |
| **2** | Leg pain | ⓪ | **①** | **②** | **③** | **④** |
| **3** | Pain due to falls or injuries | ⓪ | **①** | **②** | **③** | **④** |

| **DEMOGRAPHICS** | | | |
| --- | --- | --- | --- |
| Please fill in the blanks and mark √ one box. | | | |
| **Gender of**  **your child** | **□ Male □ Female** | **Date of birth** | **year month day** |
| **Respondent** | **□ Mother □ Father □ Grandmother □ Grandfather**  **□ Other (please specify: )** | | |
| **Age of**  **respondent** | **years** | **Education of**  **respondent** | **□ post-graduate degree**  **□ college graduate**  **□ high school graduate**  **□ lower than middle school** |
